# Comprehensive characterization of long QT syndrome‐associated genes in cancer and development of a robust prognosis model

**DOI:** 10.1111/jcmm.70094

**Published:** 2024-09-24

**Authors:** Jincheng Xu, Zhengchao Wen, Yongtao She, Maohao Li, Xiuyun Shen, Fengnan Zhi, Shu Wang, Yanan Jiang

**Affiliations:** ^1^ Department of Pharmacology (National Key Laboratory of Frigid Zone Cardiovascular Diseases, the State‐Province Key Laboratories of Biomedicine‐Pharmaceutics of China, Key Laboratory of Cardiovascular Research, Ministry of Education), College of Pharmacy Harbin Medical University Harbin China; ^2^ Cardiology Department The First Affiliated Hospital of Harbin Medical University Harbin China; ^3^ College of Bioinformatics Science and Technology Harbin Medical University Harbin China; ^4^ Translational Medicine Research and Cooperation Center of Northern China Heilongjiang Academy of Medical Sciences Harbin China

**Keywords:** biomarker, cancer, long QT syndrome, prognosis

## Abstract

Cancer is the leading public health problem worldwide. However, the side effects accompanying anti‐cancer therapies, particularly those pertaining to cardiotoxicity and adverse cardiac events, have been the hindrances to treatment progress. Long QT syndrome (LQTS) is one of the major clinic manifestations of the anti‐cancer drug associated cardiac dysfunction. Therefore, elucidating the relationship between the LQTS and cancer is urgently needed. Transcriptomic sequencing data and clinic information of 10,531 patients diagnosed with 33 types of cancer was acquired from TCGA database. A pan‐cancer applicative gene prognostic model was constructed based on the LQTS gene signatures. Meanwhile, transcriptome data and clinical information from various cancer types were collected from the GEO database to validate the robustness of the prognostic model. Furthermore, the expression level of transcriptomes and multiple clinical features were integrated to construct a Nomo chart to optimize the prognosis model. The ssGSEA analysis was employed to analysis the correlation between the LQTS gene signatures, clinic features and cancer associated signalling pathways. Our findings revealed that patients with lower LQTS gene signatures enrichment levels exhibit a poorer prognosis. The correlation of enrichment levels with the typical pathways was observed in multiple cancers. Then, based on the 17 LQTS gene signatures, we construct a prognostic model through the machine‐learning approaches. The results obtained from the validation datasets and training datasets indicated that our prognostic model can effectively predict patient outcomes across diverse cancer types. Finally, we integrated this model with clinical features into a nomogram, demonstrating its potential as a valuable prognostic tool for cancer patients. Our study sheds light on the intricate relationship between LQTS and cancer pathways. A LQTS feature based clinic decision tool was developed aiming to enhance precision treatment of cancer.

## 1 INTRODUCTION

Cancer remains a major public health challenge worldwide, responsible for nearly 10 million deaths in 2020.^1^ In 2023, there is an estimated 1,958,310 new cancer cases and 609,820 cancer deaths in the United States.^2^ Therefore, it is still an urgent need to identify novel diagnostic biomarkers and therapeutic targets for cancer. The reasonable use of antitumor drugs stands as a pivotal aspect in the comprehensive treatment of cancer and decides the prognosis of patients.^3^, ^4^, ^5^ Nevertheless, the cardiotoxicity and adverse cardiac events triggered by certain anti‐cancer agents have constrained their clinical application.

Long QT syndrome (LQTS) is a kind of cardiac electrophysiological disorder characterized by a prolongation of the corrected QT (QTc) interval on electrocardiographic recordings. This syndrome frequently emerges as a cardiac complication in cancer patients undergoing treatment, posing a significant risk for the development of symptomatic ventricular arrhythmias and a heightened likelihood of sudden cardiac death. Notably, a range of antitumor drugs, including arsenic trioxide, pazopanib, sunitinib, and imatinib, have been implicated in causing LQTS, as reported in numerous studies.^6^, ^7^, ^8^ The incidence of LQTS varied between 0% and 22% in patients receiving conventional medical therapy, between 0% and 22.7% in those receiving targeted therapy, and from 0% to 5.2% in patients with severe prolongation.^9^

Congenital LQTS represents a leading cause of sudden death among the young individuals, and without adequate treatment, LQTS patients face mortality rates as high as 21%.^10^ Pathogenic variants in at least 17 genes have been identified in patients with congenital LQTS. It is estimated that 75%–80% of congenital LQTS are caused by pathogenic variations in either KCNQ1‐encoded Kv7.1 (LQT1), KCNH2‐encoded Kv11.1 (LQT2), or SCN5A‐encoded Nav1.5 (LQT3). Acquired LQTS usually results from QT‐prolonging medications, especially anti‐cancer drugs. These medications have the potential to modulate the expression of LQTS‐associated genes. For instance, tyrosine kinase inhibitors such as crizotinib and nilotinib can induce LQTS through inhibiting hERG current encoded by KCNH2.^11^ Arsenic trioxide‐induced LQTS was associated with the inhibition of KCNH2 and KCNQ1.^12^, ^13^

LQTS‐associated genes have been implicated in the initiation and progression of cancer. Data from TIMER2.0 database reveals that the mutation frequency of ANK2 reached 19% in LUAD and SKCM, while in UCEC, LUSC, and COAD, the mutation rate exceeded 10% of the tumour samples. The mutation frequency of KCNH2 in SKCM and UCEC surpassed 7.5%. The mutation frequency of KCNQ1 in SKCM and UCEC was over 4%. This suggests a strong correlation between LQTS‐related genes and cancer prognosis. For example, the expression of ANK2 in colorectal cancer and lung adenocarcinoma is significantly downregulated. When ANK2 is expressed, there is a noticeable inhibition of tumour cell proliferation, migration and invasion.^14^, ^15^ Patients with high expression of KCNQ1 in the TCGA‐LUAD cohort showed favourable outcomes. On the contrary, knocking down KCNQ1 was found to enhance the migration capability of lung cancer cells. While, lapatinib proved to be a preferred treatment for patients exhibiting low KCNQ1 expression.^16^ Zheng and Song^17^ analysed the role of KCNH2 across different types of cancers and found that it is differentially expressed in multiple cancers and has high diagnosis and prognosis potential. The expression of SCN5A encoded protein was significantly higher in normal colon tissues than adjacent normal tissues. The knockdown of SCN5A inhibited the invasive ability of colon cancer.^18^ Although significant strides have been made, the role of LQTS‐associated genes in cancer still need further comprehensively characterized.

In this study, we integrated all 17 LQTS‐associated gene signatures and analysed their correlations across 33 cancer types. Utilizing the LASSO‐COX regression machine‐learning method, we constructed a prognostic model. Initially, we compared the prognostic ability of the model for patients with various cancers at the transcriptional level. Subsequently, we systematically integrated 83 cancer‐related pathways and compare the differences in pathway activity between the high‐risk and low‐risk groups, thus verifying the stability and reliability of the model. To further enhance clinical decision‐making ability in cancer treatment, we developed a nomogram that integrated the model and clinical features. In comparison to other commonly used prognostic models and clinical methods, our nomogram exhibited superior prognostic accuracy. This clinical decision‐making tool offers potential application in multiple cancer treatments. Our study marks a significant advancement in understanding the relationship between LQTS and cancer, bridging the research gap regarding the impact of LQTS‐related genes on cancer progression.

## 2 MATERIALS AND METHODS

### 2.1 Data sources

For the model construction, the transcriptome data and clinical information of training cohort were derived from the Cancer Genome Atlas (TCGA) database,^19^ download from UCSC database. The validation datasets were obtained from GEO and EBI database, including GSE10846, GSE26936, GSE76427, GSE42568, GSE33371, GSE41613 and E‐MTAB‐1980 cohort. A total of 33 cancer types were included in this study (Table 1), and 17 LQTS‐associated genes (Table 2) were included for follow‐up studies.

**TABLE 1:** Abbreviations list of enrolled cancer types.

Abbreviation	Full name
ACC	Adrenocortical carcinoma
BLCA	Bladder Urothelial Carcinoma
BRCA	Breast invasive carcinoma
CESC	Cervical squamous cell carcinoma and endocervical adenocarcinoma
CHOL	Cholangiocarcinoma
COAD	Colon adenocarcinoma
DLBC	Lymphoid Neoplasm Diffuse Large B‐cell Lymphoma
ESCA	Oesophageal carcinoma
GBM	Glioblastoma multiforme
HNSC	Head and Neck squamous cell carcinoma
KICH	Kidney Chromophobe
KIRC	Kidney renal clear cell carcinoma
KIRP	Kidney renal papillary cell carcinoma
LGG	Brain Lower Grade Glioma
LIHC	Liver hepatocellular carcinoma
LUAD	Lung adenocarcinoma
LUSC	Lung squamous cell carcinoma
MESO	Mesothelioma
OV	Ovarian serous cystadenocarcinoma
PAAD	Pancreatic adenocarcinoma
PCPG	Pheochromocytoma and Paraganglioma
PRAD	Prostate adenocarcinoma
READ	Rectum adenocarcinoma
SARC	Sarcoma
SKCM	Skin Cutaneous Melanoma
STAD	Stomach adenocarcinoma
TGCT	Testicular Germ Cell Tumours
THCA	Thyroid carcinoma
THYM	Thymoma
UCEC	Uterine Corpus Endometrial Carcinoma
UCS	Uterine Carcinosarcoma
UVM	Uveal Melanoma

**TABLE 2:** The list of 17 LQTS‐associated genes.

Gene
KCNQ1
KCNH2
SCN5A
ANK2
KCNE1
KCNE2
KCNJ2
CACNA1C
CAV3
SCN4B
AKAP9
SNTA1
KCNJ5
CALM1
CALM2
CALM3
TRDN

### 2.2 Analysis of expression pattern of 17 LQTS‐associated genes across cancer types

Based on the TCGA transcriptome data, we mapped the differential expression status of 17 LQTS‐associated genes in tumours and non‐tumours. We then used differential expression bubble maps to demonstrate the differential expression of these 17 genes across 19 cancer types. |log2FC| ≥ 1 and *p* < 0.05 was considered statistically significant.

### 2.3 Prognosis analysis of 17 LQTS‐associated genes

The correlation between the expression of LQTS‐associated genes and the prognosis of different cancers were analysed using the data from TCGA database and GEO database. The correlation between gene expression level and the overall survival (OS), disease specific survival (DSS) and progression free interval (PFI) were analysed according to the best cut‐off value using the ‘survival’ and ‘survminer’ R packages. RNAseq data from TCGA and GEO was analysed and visualized using the ‘timeROC’ and ‘ggplot2'software package. The area under the ROC curves (AUC) was calculated for the evaluation of prognosis prediction value of genes.

### 2.4 Enrichment status of LQTS‐associated genes

Enrichment status of LQTS‐associated gene signatures and activity of the cancer associated signalling pathway were evaluated using the R package ‘GSVA’.^20^, ^21^

### 2.5 Feature selection by LASSO regression

In this study, a combination of candidate genes associated with LQTS was selected using the features of LASSO feature selection. LASSO regression analysis was performed using the R package glmnet.^22^

### 2.6 Development and validation of nomograph

A nomograph was constructed via the R package ‘rms’. In this way, we merge the risk score with clinicopathological features, such as age, gender, and stage to develop a comprehensive clinic prognosis indicator. The accuracy of the forecast was evaluated via a correction chart, which illustrated the variance between forecasted and real survivorship, with the 45‐degree line being the ideal forecast outcome.

### 2.7 Statistical analysis

Continuous variables were compared by Wilcoxon test and Student's *t*‐test between two groups and by Kruskal–Wallis test between multiple groups. Correlation between two continuous variables was analysed by Spearman correlation.

## 3 RESULTS

### 3.1 The expression and diagnostic ability of LQTS‐associated genes in cancer

**FIGURE 1:**
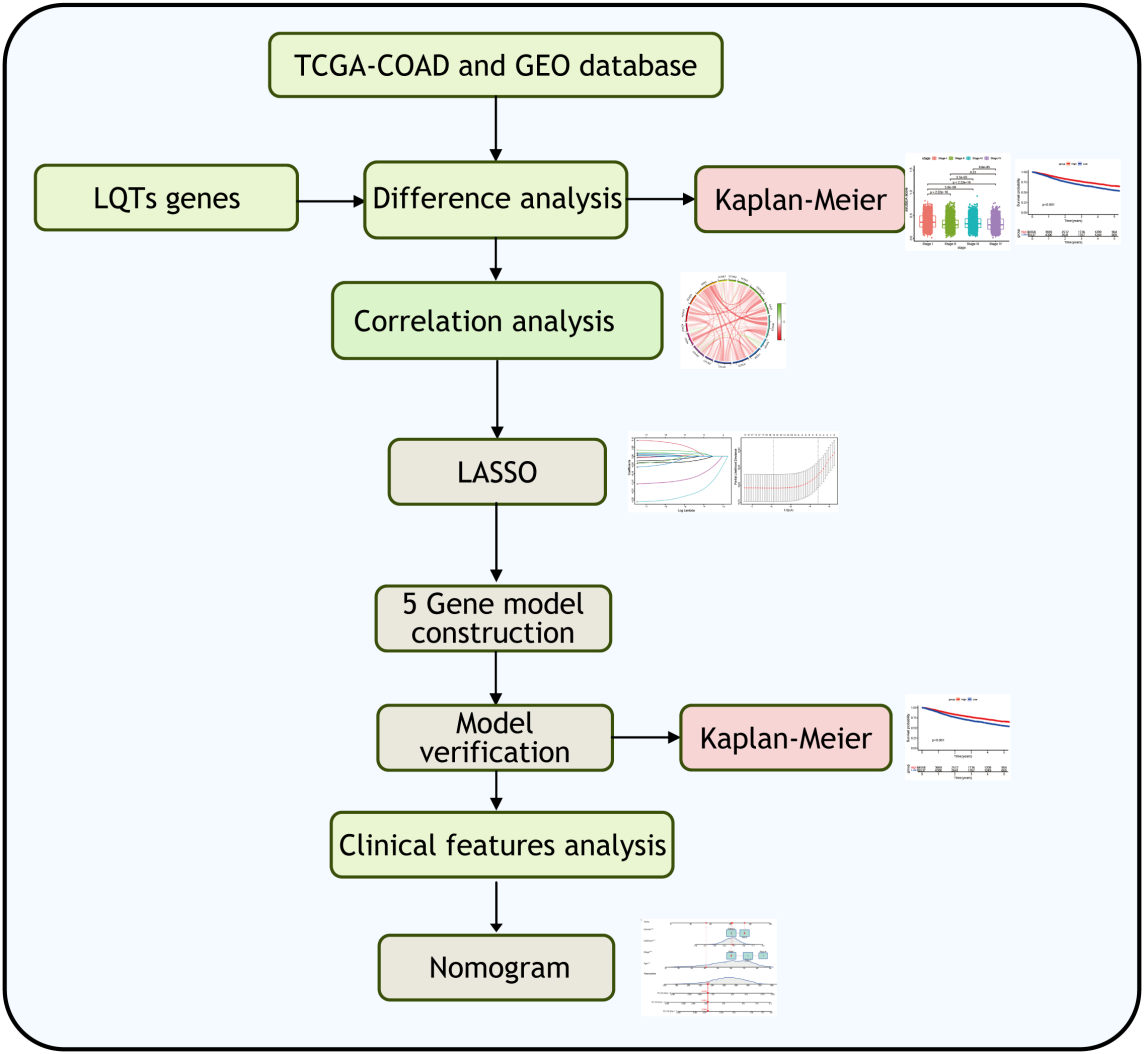
The workflow of this study. Analysis of LQTS‐associated genes in cancer was performed using data in the TCGA and GEO databases.

**FIGURE 2:**
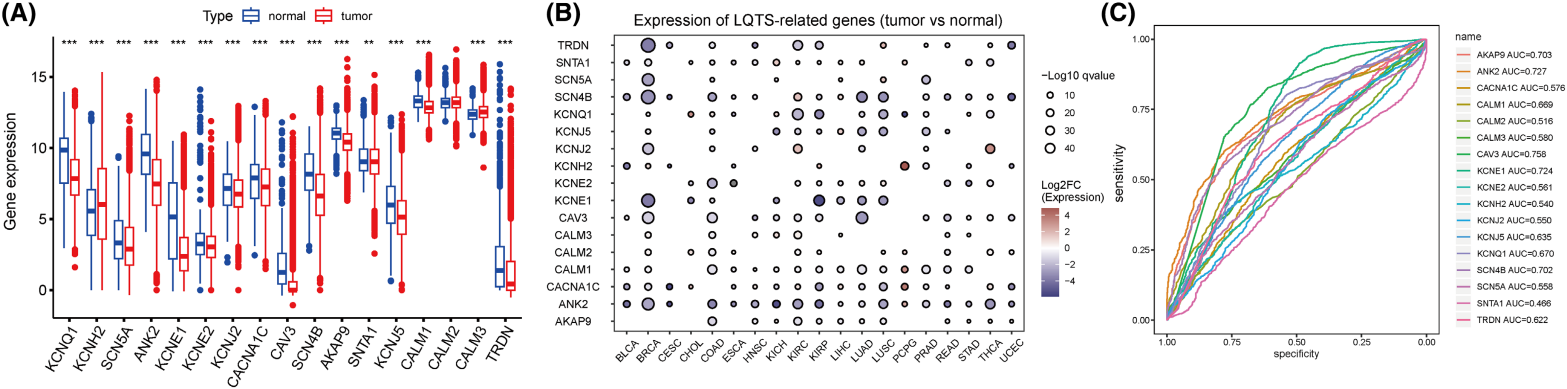
The expression and prognostic value of LQTS‐associated genes in cancers. (A) The expression of long QT syndrome genes in cancer. (B) Bubble map of differential expression of 17 long LQTS‐associated genes between tumour and adjacent normal tissue. (C) The ROC curve and AUC value of long QT syndrome genes in cancer.

The workflow of this study was shown in Figure 1. The expression levels of 17 LQTS‐associated genes in pan‐cancer were calculated. Among them, KCNH2 and CALM3 were highly expressed in the tumour group, KCNQ1, SCN5A, ANK2, KCNE1, KCNE2, KCNJ2, CACNA1C, CAV3, SCN4B, AKAP9, SNTA1, KCNJ5, CALM1, CALM2 and TRDN was highly expressed in the normal group (Figure 2A). The bubble map showed that the expression of LQTS‐associated genes was significantly downregulated in the tumour group compared with the normal group (Figure 2B). Then, we evaluated the diagnostic potential of LQTS‐related gene expression in distinguishing between tumour patients and healthy individuals. The AUC value for 5 genes was greater than 0.7, including CAV3, ANK2, KCNE1, AKAP9, and SCN4B. And, the AUC value for 4 genes were between 0.6 and 0.7, including KCNQ1, CALM1, KCNJ5, and TRDN (Figure 2C).

### 3.2 The predictive efficiency of LQTS‐associated genes in cancer prognosis

**FIGURE 3:**
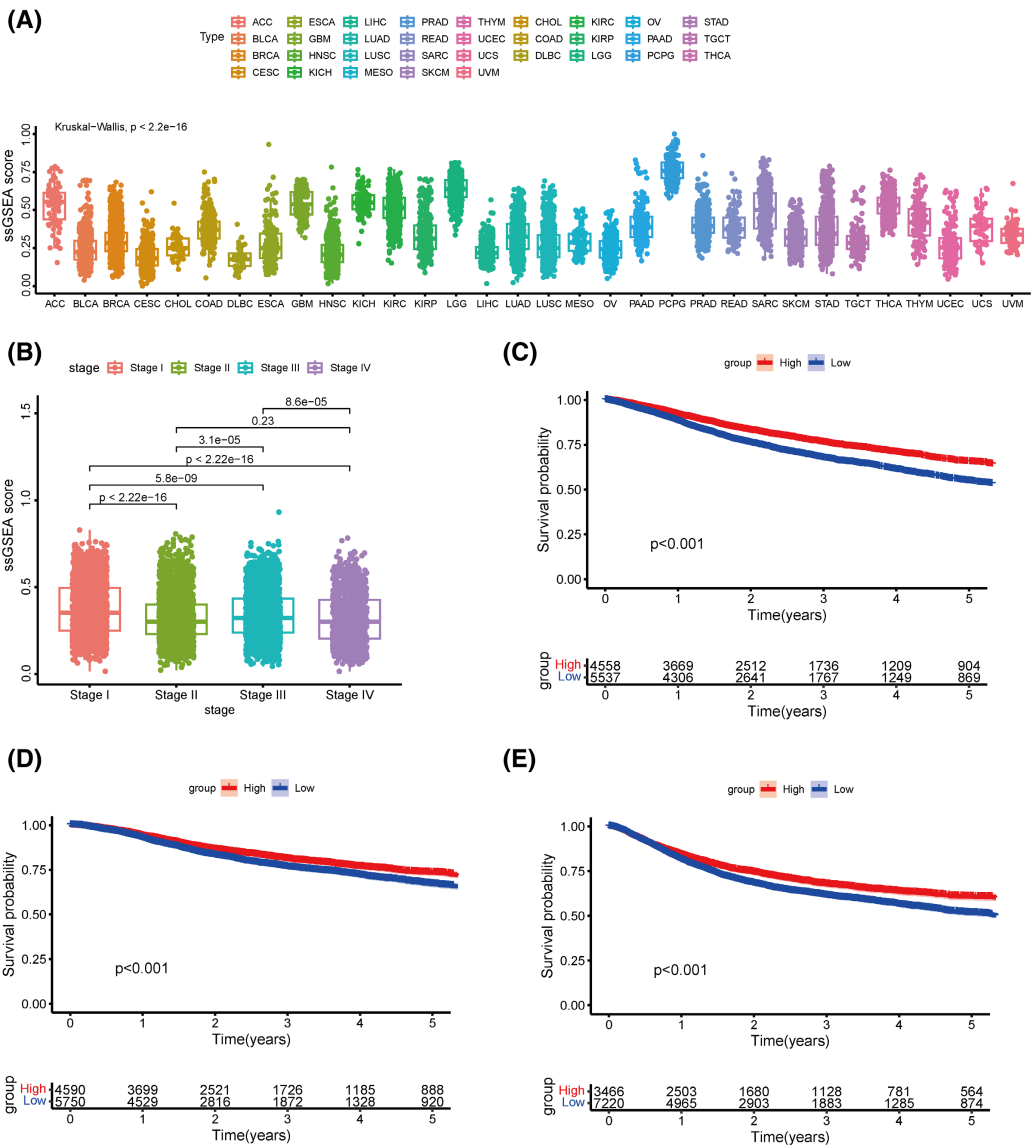
Correlation between the enrichment levels of LQTS gene and prognosis of cancer. (A) There were significant differences in the enrichment levels of LQTS‐associated genes in cancer. (B) Correlation between enrichment levels of LQTS‐associated genes and cancer stage. (C–E) Kaplan–Meier analysis of the association between enrichment levels of long QT syndrome genes and OS (C), DSS (D), PFI (E).

We evaluated the enrichment status of the LQTS‐associated genes in cancer. The results revealed that significant heterogeneity in the enrichment levels of LQTS‐ associated genes across different cancer types (Figure 3A). Additionally, we observed a notable decrease in the ssGSEA score as the tumour stage progressed, suggesting a reduction in the activity of LQTS‐related genes. This finding consistent with our earlier observations that LQTS‐related genes are predominantly underexpressed in tumours (Figure 3B). Subsequently, we analysed the association between the enrichment level of LQTS‐related genes and prognosis of cancer. A high enrichment level of LQTS‐associated genes was correlated with improved OS, DSS, and PFI (Figure 3C–E).

### 3.3 Association between expression level of LQTS‐associated genes and cancer associated signalling pathway

**FIGURE 4:**
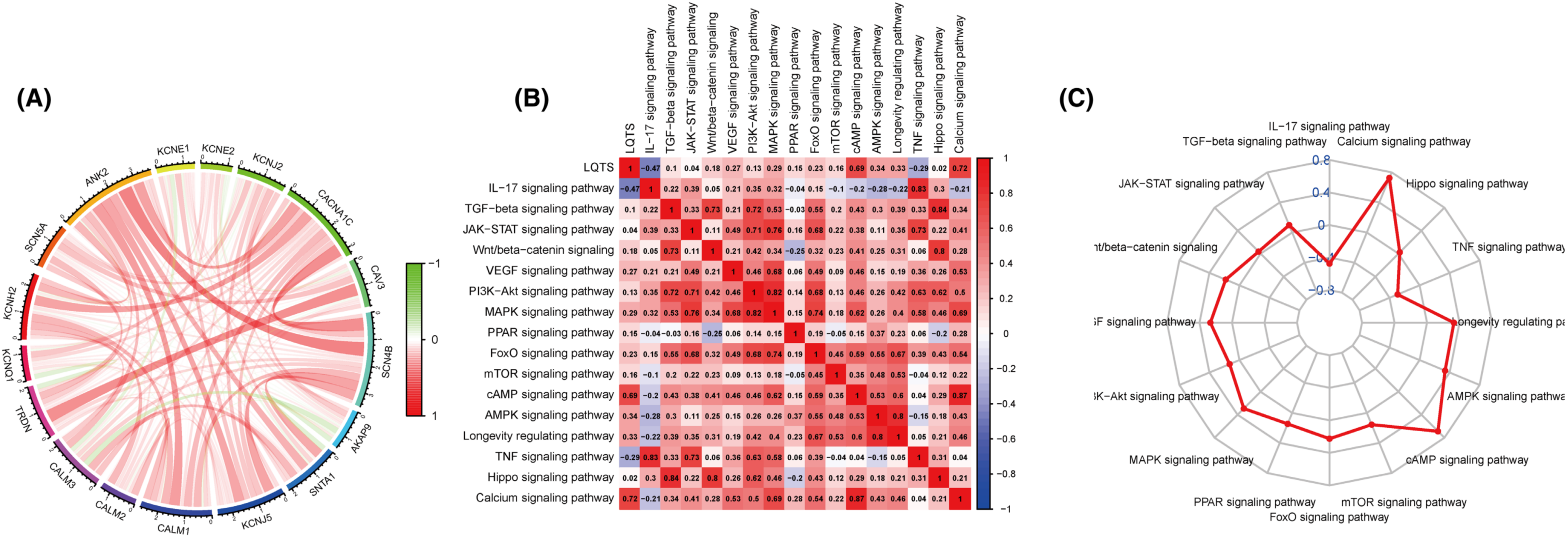
Correlation between the enrichment levels of LQTS‐associated genes and cancer associated signalling pathway. (A) Correlation map of 17 long QT syndrome genes. (B and C) Heat and radar maps demonstrate the correlations between long QT syndrome genes and cancer associated signalling pathway.

The correlation circles of 17 LQTS‐associated genes depicted a predominantly positive linkage among these genetic markers (Figure 4A). To further delineate the relationship between these genes and cancer biology, we retrieved 16 cancer‐signalling pathways from the KEGG database. Subsequently, a thorough analysis was conducted to explore the association between LQTS‐associated genes and these critical signalling pathways. The results revealed a robust association between LQTS‐associated genes and cancer signalling pathways, including cAMP, IL‐17, and AMPK signalling pathways (Figure 4B,C).

### 3.4 Construction of a prognostic model based on LQTS‐associated genes

**FIGURE 5:**
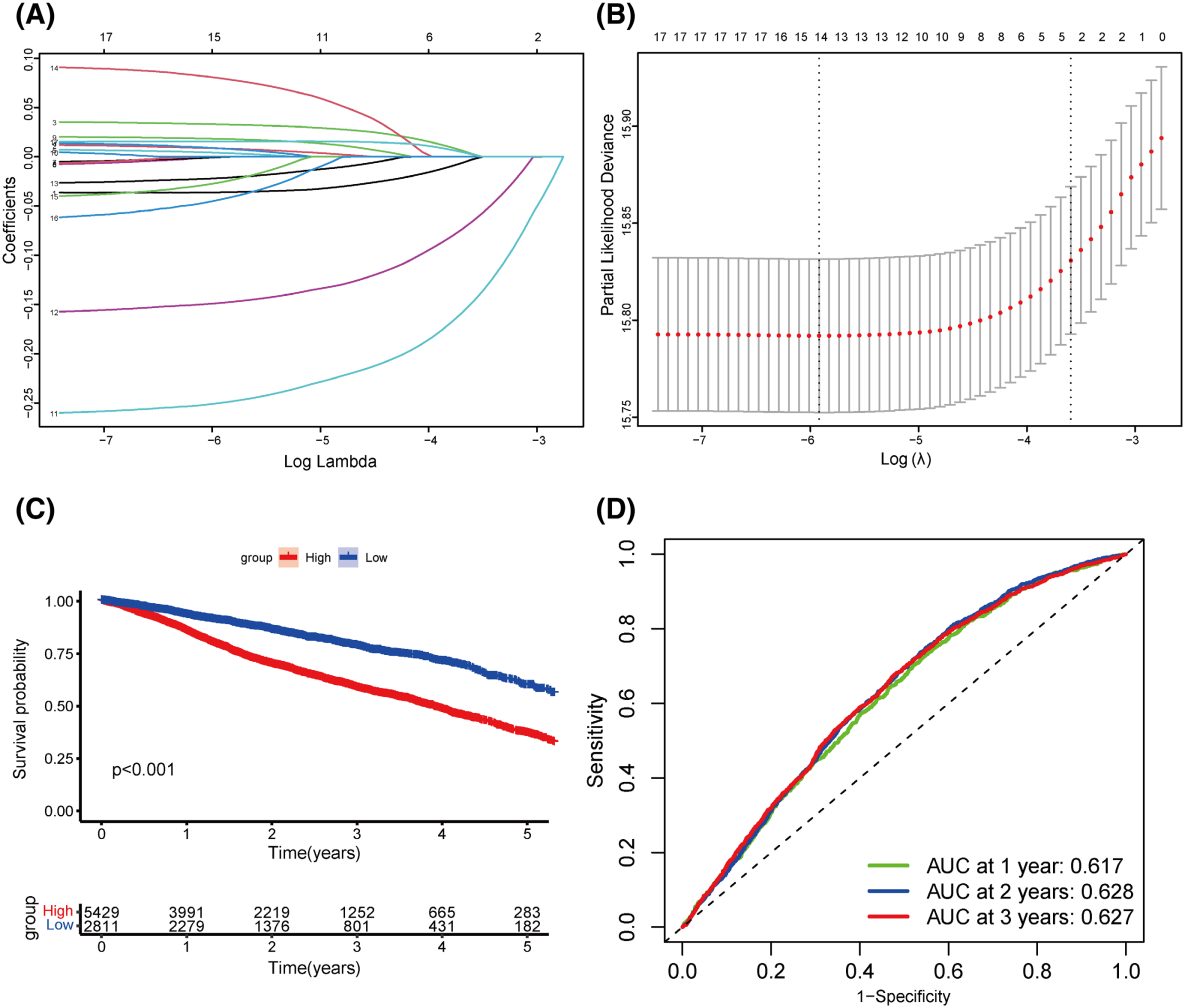
A LQTS gene‐based model for prognosis of cancer. (A and B) The coefficients calculated by multivariate Cox regression using LASSO‐COX regression. (C) Kaplan–Meier overall survival (OS) curves for pan‐cancer patients. (D) AUC curve of the prognostic model for pan‐cancer patients.

To explore the prognostic potential of LQTS‐associated genes, we employed LASSO‐COX regression analysis to constructed a prognosis model. A five‐gene (KCNQ1, SCN5A, AKAP9, SNTA1, TRDN) involved prognostic model was constructed (Figure 5A,B). Using this model, patients were categorized into high and low LQTS score groups based on a predefined cut‐off value. Patients in the low LQTS score group exhibited better OS than those in the high LQTS score group (Figure 5C). Furthermore, ROC curve analysis showed that the AUC values of 1, 2, and 3 years of total survival were 0.617, 0.628, 0.627, respectively (Figure 5D).

### 3.5 Survival analysis of the constructed prognostic model in cancer patients

**FIGURE 6:**
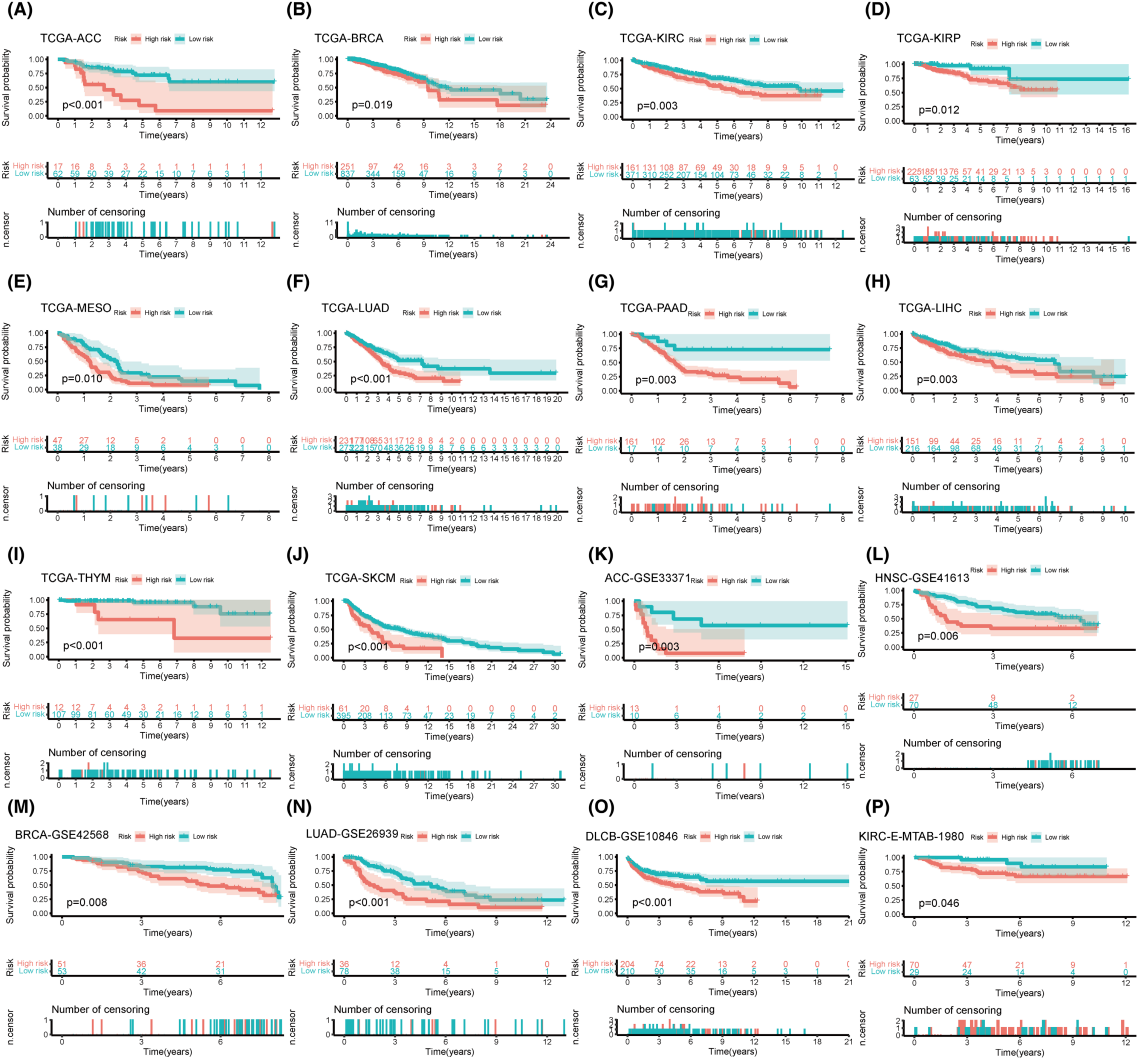
Survival analysis of prognostic models in multiple cancer types. Overall survival (OS) analysis of Prognostic model in TCGA dataset and other verification set by Kaplan–Meier curve. (A–J) TCGA cohorts, ACC (A), BRCA (B), KIRC (C), KIRP (D), MESO (E), LUAD (F), PAAD (G), LIHC (H), THYM(I), SKCM(J). (K–O) GEO cohorts, ACC (K), HNSC (L), BRCA (M), LUAD (N), DLCB (O). N: E‐MTAB‐1980 cohort, KIRC (P).

We further analysed the prognostic implications of our model in specific cancers. For TCGA cohorts, the outcomes of patients in the low LQTS score group was better than that in the high LQTS score group (Figure 6A–J). To further validate the prognostic value of our model, it was verified in five GEO datasets (Figure 6K–O) and E‐MTAB‐1980 cohort (Figure 6P). Consistent with our initial findings, the results revealed that patients with lower LQTS scores exhibited significantly better outcomes. Moreover, we undertook an additional evaluation of the protein expression of the KCNQ1 gene based on immunohistochemical images in the Human Protein Atlas (HPA) database.^23^ It was observed that the protein expression of KCNQ1 gene in 8 cancers was significantly lower than that in normal tissues (Figure S1).

### 3.6 The correlation between risk score and common clinical measures

**FIGURE 7:**
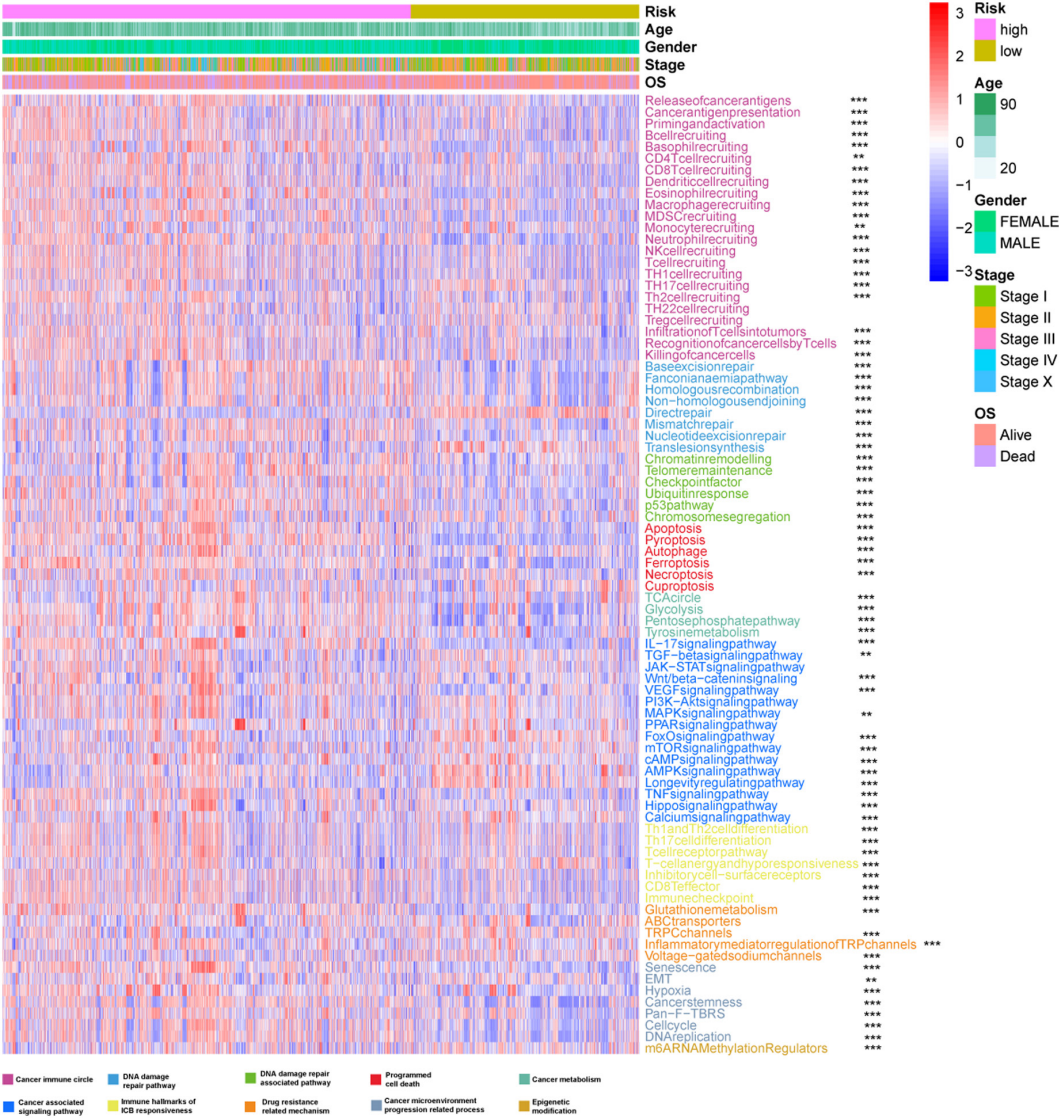
Differences in tumour‐related pathway activity in different groups of the TCGA cohort. The 83 pathway‐related functions are displayed in different colours at the bottom of the heat map. The biology process activity shows the activity scores after standardization of 83 pathways. Stage, Gender, OS, and Age are different clinical indicators of patients in the pan‐cancer cohort.

**FIGURE 8:**
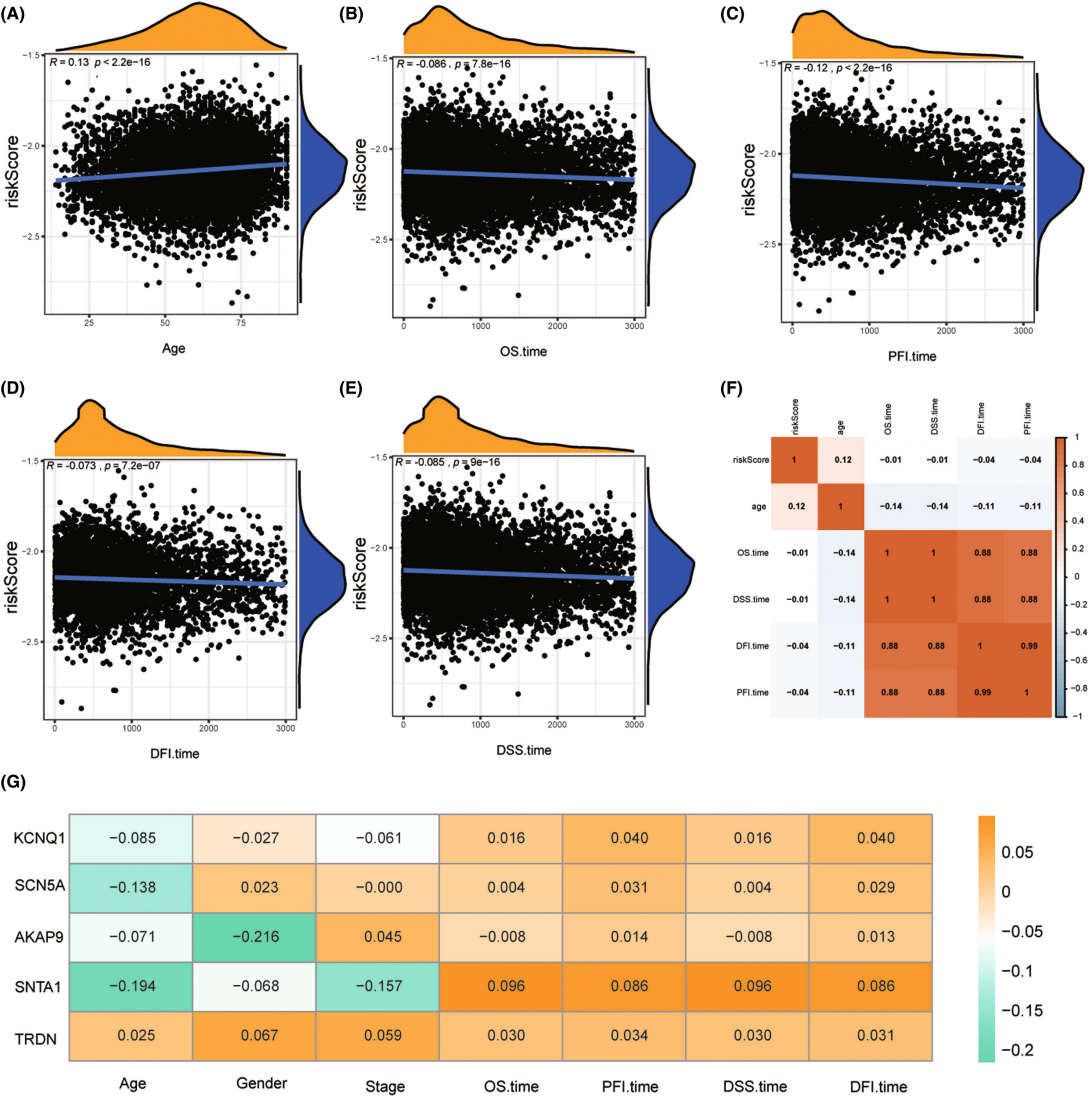
Correlation analysis between risk score and different clinical indicators. (A) Correlation of risk score with Age. (B) Correlation of risk score with OS time. (C) Correlation of risk score with PFI time. (D) Correlation of risk score with DFI time. (E) Correlation of risk score with DSS time. (F) Heat map of correlation between risk score and relevant clinical indicators. (G) Prognosis of LQTS genes and clinical features of correlation.

We then investigate the association between risk scores and common clinical features of cancer. We obtained 83 cancer‐related pathways from the literature and KEGG, and visualized their GSVA scores using heat maps. The results showed that cancer‐related pathways displayed increased activity in the patients from majority of high‐risk groups (Figure 7 and Figure S2). The detailed P‐values for the pathways depicted in the heat map are listed in Table 3. Subsequently, we analysed the correlation of risk score and age, OS, PFI, DFI, and DSS (Figure 8A–F). These results showed that the risk score was positively correlated with age and negatively correlated with other mentioned indicators.

**TABLE 3:** The detailed *p*‐values of the 83 cancer‐related pathways.

Pathway	*p*
Release of cancer antigens	2.22E‐57
Cancer antigen presentation	1.94E‐40
Priming and activation	1.73E‐49
B‐cell recruiting	2.50E‐57
Basophil recruiting	6.34E‐125
CD4 T‐cell recruiting	0.001490335
CD8 T‐cell recruiting	3.84E‐29
Dendritic cell recruiting	4.56E‐64
Eosinophil recruiting	6.34E‐125
Macrophage recruiting	2.37E‐36
MDSC recruiting	8.07E‐131
Monocyte recruiting	0.001828572
Neutrophil recruiting	1.67E‐180
NK cell recruiting	4.72E‐41
T‐cell recruiting	2.36E‐46
TH1 cell recruiting	4.22E‐43
TH17 cell recruiting	2.13E‐05
Th2 cell recruiting	5.25E‐39
TH22 cell recruiting	0.638713792
Treg cell recruiting	0.580157765
Infiltration of T cells into tumours	1.70E‐28
Recognition of cancer cells by T cells	5.34E‐68
Killing of cancer cells	7.33E‐42
Base excision repair	3.72E‐128
Fanconi anaemia pathway	3.55E‐72
Homologous recombination	4.77E‐50
Non‐homologous end joining	1.27E‐11
Direct repair	1.42E‐228
Mismatch repair	8.61E‐43
Nucleotide excision repair	8.94E‐77
Translesion synthesis	4.85E‐57
Chromatin remodelling	9.19E‐19
Telomere maintenance	6.76E‐86
Checkpoint factor	3.16E‐32
Ubiquitin response	2.84E‐93
p53 pathway	3.90E‐101
Chromosome segregation	2.27E‐06
Apoptosis	7.59E‐138
Pyroptosis	0
Autophage	5.13E‐10
Ferroptosis	7.10E‐261
Necroptosis	6.80E‐27
Cuproptosis	0.086101619
TCA circle	2.95E‐25
Glycolysis	1.55E‐130
Pentose phosphate pathway	1.27E‐176
Tyrosine metabolism	1.66E‐35
IL‐17 signaling pathway	2.23E‐203
TGF‐beta signaling pathway	0.00991135
JAK‐STAT signaling pathway	0.094145233
Wnt/beta‐catenin signaling	8.49E‐14
VEGF signaling pathway	2.73E‐48
PI3K‐Akt signaling pathway	0.066809929
MAPK signaling pathway	0.003428209
PPAR signaling pathway	0.610209768
FoxO signaling pathway	5.27E‐87
mTOR signaling pathway	1.94E‐12
cAMP signaling pathway	7.48E‐11
AMPK signaling pathway	6.47E‐42
Longevity regulating pathway	8.01E‐69
TNF signaling pathway	1.37E‐71
Hippo signaling pathway	9.05E‐17
Calcium signaling pathway	3.14E‐06
Th1 and Th2 cell differentiation	5.66E‐17
Th17 cell differentiation	2.84E‐11
T‐cell receptor pathway	2.00E‐24
T‐cell anergy and hyporesponsiveness	4.56E‐09
Inhibitory cell‐surface receptors	9.39E‐39
CD8T effector	8.33E‐52
Immune checkpoint	6.13E‐45
Glutathione metabolism	2.14E‐142
ABC transporters	0.318712849
TRPC channels	5.74E‐45
Inflammatory mediator regulation of TRP channels	0.000136514
Voltage‐gated sodium channels	2.95E‐23
Senescence	4.05E‐191
EMT	0.004055229
Hypoxia	6.58E‐60
Cancer stemness	2.38E‐149
Pan‐F‐TBRS	2.48E‐41
Cell cycle	8.56E‐122
DNA replication	1.53E‐171
m6ARNA methylation regulators	0.000235886

### 3.7 Development and validation of a nomograph for predicting OS

**FIGURE 9:**
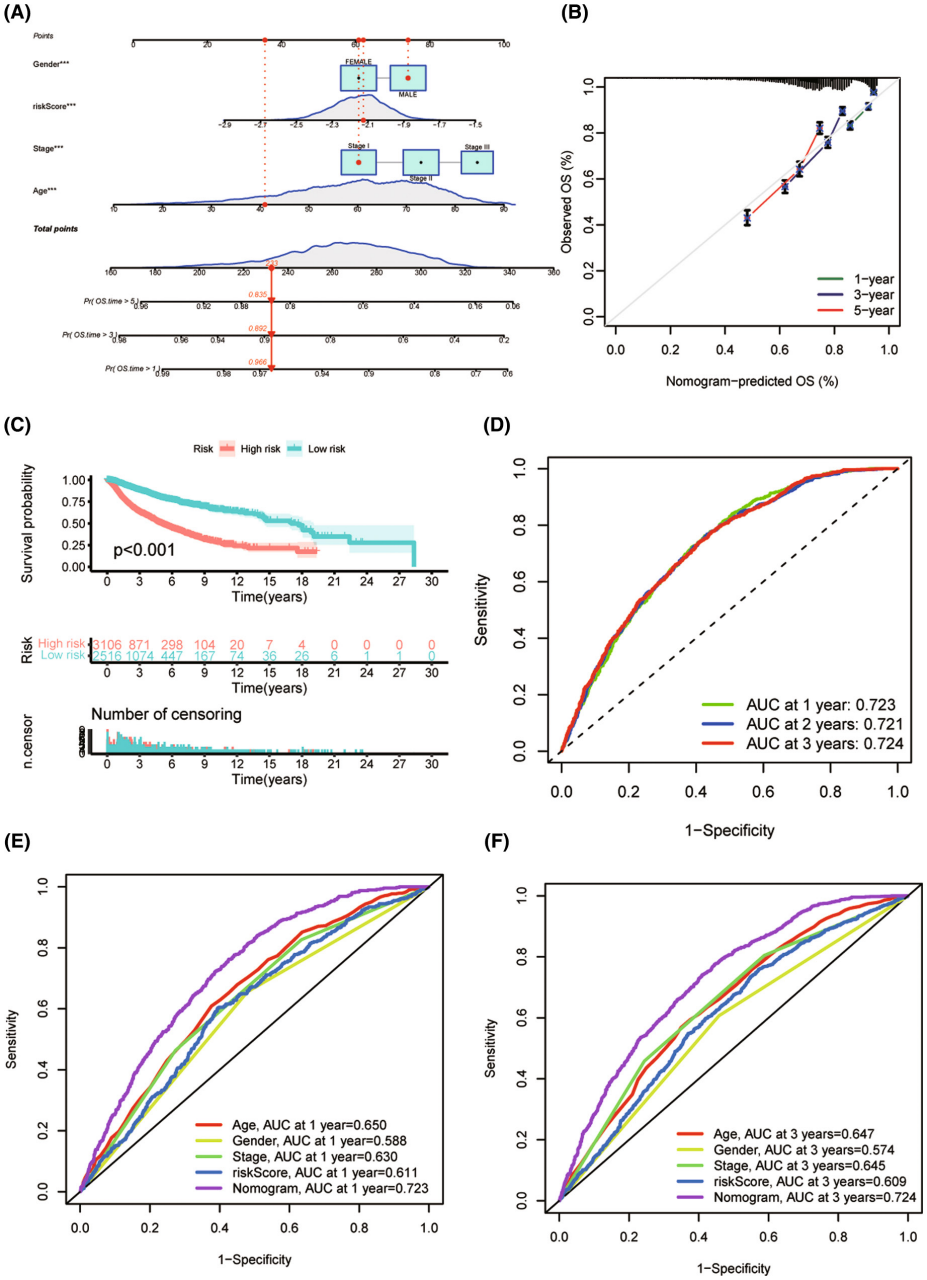
Establishment and prognostic value of nomogram based on prognostic model of LQTS‐associated genes. (A) Nomograph on the basis of LQTS score can predict 1, 3 and 5 year survivorship in pan‐cancer patients. (B) Nomograph calibration curves for predicting 1‐, 3‐, and 5‐year survivorship. (C) Kaplan–Meier curve showing a comparison of OS among low‐ and high‐risk groups. (D) AUC curve of the nomograph. (E) AUC values of nomograms and other clinical diagnostic features for predicting 1‐year survival times. (F) AUC values of nomograms and other clinical diagnostic features for predicting 3‐year survival times.

Based on the risk score and clinicopathological characteristics, we developed a nomograph to predict OS at the 1, 3, and 5 years. The scores for each variable in the nomograph can be found in the score table. The overall score was used to predict the survivorship possibility at the 1, 3, and 5 years (Figure 9A). The predictive accuracy of the nomograph was analysed via a correction curve. The results showed that the prediction performance of our nomogram was favourable (Figure 9B). The prognosis of patients in the low‐risk group was better compared with those in the high‐risk group (Figure 9C). The AUC values of nomograph signature prediction ability were 0.723, 0.721 and 0.724 at the 1, 3, and 5 years, respectively, which were better than LQTS score (Figure 9D). Next, we compared the nomograph with other diagnostic methods in cancer clinical data, and the results showed that the nomograph still had the highest accuracy (Figure 4E,F).

## 4 DISCUSSION

Cancer remains one of the leading causes of death worldwide.^24^ Even though, reasonable application of antitumor drugs has the potential to extend patients survival. However, a major concern with most antitumor drugs is their propensity to induce serious side effects, particularly cardiotoxicity.^25^, ^26^ A common clinical manifestation of cardiotoxicity is acquired LQTS. Onco‐cardiology is an emerging field that is the intersection of cardiovascular and cancer healthcare system. Increasing studies have highlighted the correlation between LQTS and cancer.^27^ In this study, we comprehensively analysed the role of LQTS‐associated genes in cancer.

The data of cancer patients was obtained from TCGA and GEO databases. Initially, we calculated the enrichment levels and scores of LQTS‐related genes using the R software package GSVA and subsequently explored their correlation with clinical characteristics. It was found that the GSVA score was significantly correlated with the stage, OS, DSS and PFI of cancer patients. Patients in the high score group exhibited a more favourable prognosis than those in the low score group. By comparing the enrichment levels of LQTS‐associated genes with cancer associated signalling pathway, we also found that LQTS‐associated genes are closely related to most cancer signalling pathways. For example, the IL‐17 signalling pathway, known to promote cancer development, was found to be inversely correlated with LQTS.^28^ The cAMP signalling pathway plays a inhibitive role in cancer by regulating cell proliferation and apoptosis, and was positively correlated with LQTS‐associated genes.^29^ These findings were consistent with our previous findings that patients with lower LQTS enrichment levels have a better prognosis.

Through LASSO‐COX regression analysis, five genes were identified have prominent prognostic potential, including KCNQ1, SCN5A, AKAP9, SNTA1, and TRDN. Some of these genes have been reported play important role in cancer. Among them, KCNQ1 has been implicated as a potential marker in gastric cancer, colon cancer, and breast cancer.^30^, ^31^, ^32^, ^33^ SCN5A is expressed in various cancer types such as breast cancer, prostate cancer, colon cancer, and cervical cancer.^18^, ^34^, ^35^ Mutation of AKAP9 is closely related to the high microsatellite instability of gastric and colorectal cancers.^36^ AKAP9 can act as an oncoprotein to promote the progression of gastric cancer.^37^ SNTA1 derived signalling pathway can modulate the migratory potential of breast cancer.^38^ The role of TRDN in cancer still need to investigate.

Then, we constructed a LQTS score based on the coefficients of these five genes. The cancer patients were divided into high LQTS score and low LQTS score groups. In cancers such as ACC, BRCA, KIRC, KIPR, LIHC, LUAD, patients in the low‐risk group have better outcomes. By integrating the LQTS score and clinical characteristics, we developed a nomograph to predict patient OS at 1, 3, and 5 years. The nomograph calibration plots indicated that it accurately predicted the Overall survival time of patients.

The study had several limitations that will be addressed in future research. Firstly, the conclusions would have been more reliable if a large pan‐cancer cohort had been used for validation. Secondly, the addition of more data would have strengthened the reliability of the conclusions. These shortcomings will be remedied as big data continues to develop and as we conduct further research.

In conclusion, our study comprehensively characterized and highlighted the critical role of LQTS‐associated genes in cancer. These genes may be promising therapeutic targets for cancer treatment and may provide new insights into the clinical management of cancer patients combined with cardiovascular disorders. The constructed prognostic model has the potential to be applied in the decision‐making processes of multiple clinical cancer treatments.

## AUTHOR CONTRIBUTIONS

**Jincheng Xu:** Data curation (equal); formal analysis (equal); validation (equal). **Zhengchao Wen:** Formal analysis (equal). **Yongtao She:** Writing – review and editing (equal). **Maohao Li:** Formal analysis (equal). **Xiuyun Shen:** Writing – review and editing (equal). **Fengnan Zhi:** Writing – review and editing (equal). **Shu Wang:** Conceptualization (equal). **Yanan Jiang:** Conceptualization (equal); funding acquisition (equal).

## FUNDING INFORMATION

This work was supported by the Natural Science Foundation of Heilongjiang Province (Grant No. LH2021H018), the Heilongjiang Postdoctoral Foundation (Grant No. LBH‐Q21134).

## CONFLICT OF INTEREST STATEMENT

The authors have declared that no competing interest exists.

## Supporting information


Appendix S1.

## Data Availability

All original data can be acquired from the reasonable request to corresponding author‐Yanan Jiang.
