# Supplementary material for: Comprehensive characterization of long QT syndrome‐associated genes in cancer and development of a robust prognosis model
Source: J Cell Mol Med. 2024 Sep 24;28(18):e70094. doi: 10.1111/jcmm.70094 (PMC11421991; doi:10.1111/jcmm.70094)
Supplement: Supplementary file 1 — Appendix S1. [file JCMM-28-e70094-s001.zip › jcmm70094-sup-0005-Captions.docx]

**Figure S1: Expression of KCNQ1 gene in normal and tumor tissues.**

Based on HPA database, which shows the KCNQ1 gene in normal and tumor protein expression in immunohistochemical images.

**Figure S2:** **Boxplot of GSVA scores for 83 cancer-related pathways.**

**Table S1:** **Abbreviation list for the cancer types enrolled in the study.**

**Table S2:** **Statistical differences in clinical features of 83 cancer-related pathways.**
