# Supplementary figures and images for: Comprehensive characterization of long QT syndrome‐associated genes in cancer and development of a robust prognosis model
Source: J Cell Mol Med. 2024 Sep 24;28(18):e70094. doi: 10.1111/jcmm.70094 (PMC11421991; doi:10.1111/jcmm.70094)

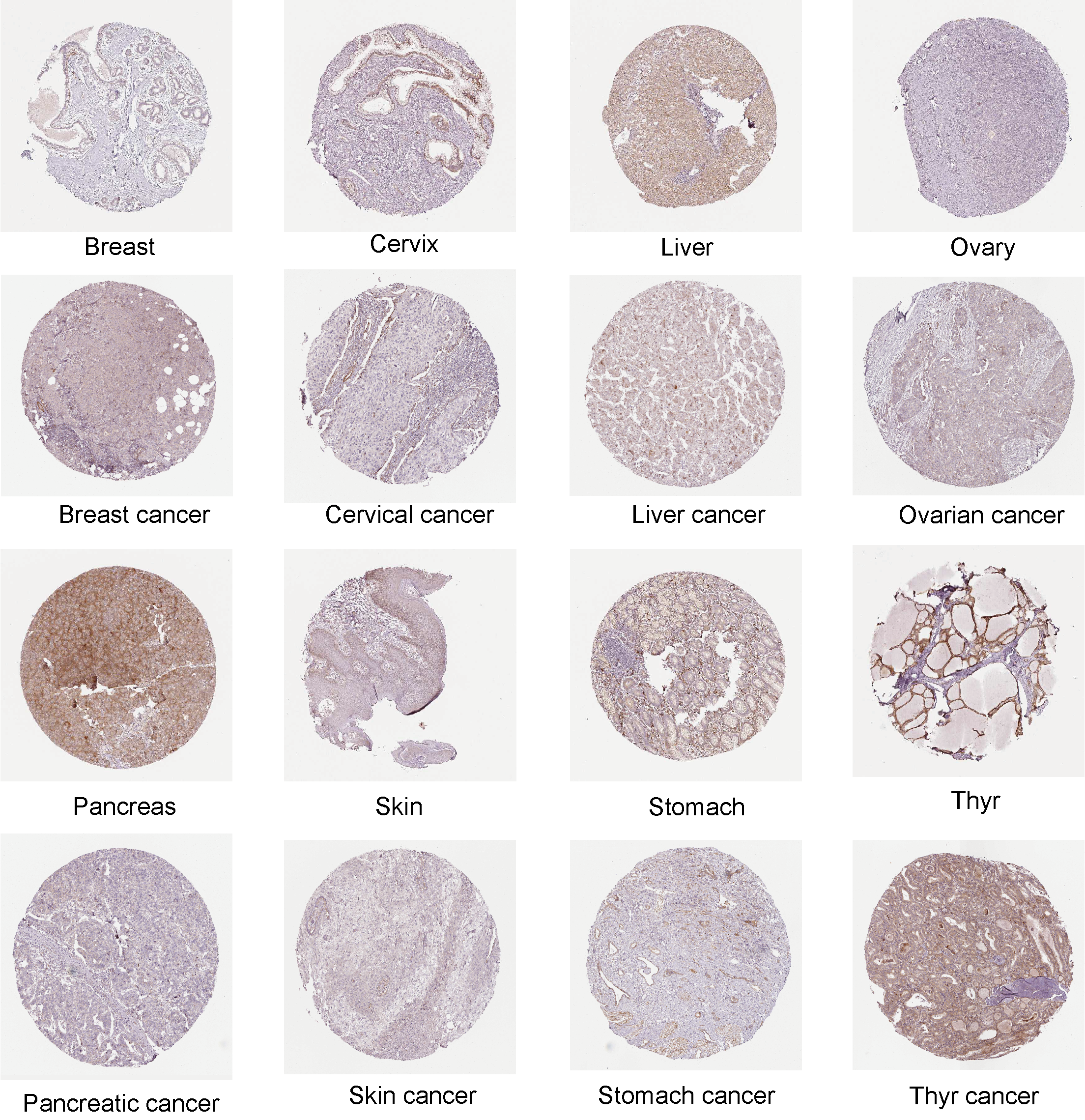

Supplement: Supplementary file 1 — Appendix S1. [file JCMM-28-e70094-s001.zip › jcmm70094-sup-0001-FigureS1.tif]

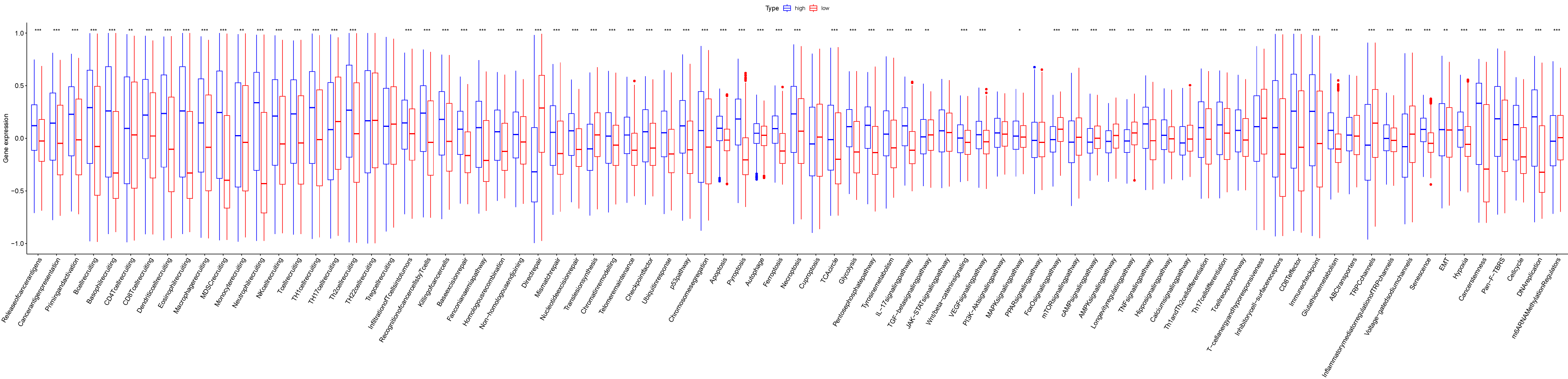

Supplement: Supplementary file 1 — Appendix S1. [file JCMM-28-e70094-s001.zip › jcmm70094-sup-0002-FigureS2.tif]
